# Ebola laboratory preparedness at frontline hospitals: can we or can’t we?

**DOI:** 10.1128/jcm.00903-26

**Published:** 2026-06-26

**Authors:** Nicholas E. Heger, Elyse Fritschel, Eliezer Nussbaum, Hunter Fraker, Justin McCallum, Beverley Orr, Robert Skahan, Elaine Minchello, Melissa Backman, Monika Pilichowska, Matthew Morrow, Malak Abedalthagafi, Zoe Freeman Weiss

**Affiliations:** 1 Department of Pathology and Laboratory Medicine, Tufts Medical Center 1867, Boston, Massachusetts, USA; 2 Infection Prevention, Tufts Medical Center 1867, Boston, Massachusetts, USA; 3 Division of Geographic Medicine, Tufts Medical Center 10624, Boston, Massachusetts, USA; Vanderbilt University Medical Center, Nashville, Tennessee, USA

**Keywords:** Ebola, viral hemorrhagic fever, laboratory biosafety

## Abstract

Frontline hospitals are required to care for patients with suspected viral hemorrhagic fever (VHF), yet guidance on laboratory preparedness remains fragmented and incomplete. We conducted a multidisciplinary risk assessment of our institutional capacity to perform routine diagnostic testing for VHF persons under investigation (PUI), focusing on the feasibility of using automated core laboratory instruments. Our assessment revealed substantial gaps between CDC guidance (which permits core lab testing) and the practical ability to implement it safely. Public health mandates for VHF preparedness have not been accompanied by granular guidance on biosafety, laboratory infrastructure, or regulatory clarity necessary for implementation. Community hospitals, which would benefit most from safely using their existing automated core laboratory instruments, lack the infrastructure, staffing expertise, and clear guidance to do so, while well-resourced tertiary centers are often best positioned to develop dedicated point-of-care testing (POCT)-based workflows. Federal and state authorities must provide explicit, validated examples of acceptable mitigation strategies for testing using core lab instrumentation and reconcile conflicting recommendations across guidance documents. Without such authoritative clarity, frontline hospitals cannot confidently meet their mandated VHF preparedness obligations.

## COMMENTARY

## BACKGROUND AND RENEWED URGENCY

In May 2026, the Ministry of Public Health of the Democratic Republic of the Congo confirmed an outbreak of Ebola disease caused by Bundibugyo virus originating in the Ituri Province. On May 17, the World Health Organization declared the outbreak a “Public Health Emergency of International Concern.” Unlike the Zaire ebolavirus, the Bundibugyo virus has no licensed vaccine or approved specific treatment (1). As of May 19, no cases have been identified in the United States and overall risk to the American public has been assessed to be low (2). Still, global challenges to containment have been noted in the setting of high population mobility, displacement in conflict-affected areas, barriers to healthcare access, and limited testing capacity (1, 3). The outbreak coincides directly with the 2026 Fédération Internationale de Football Association (FIFA) World Cup, running from June 11 to July 19, which will bring substantial international travel to North American host cities (4). Events of this scale create conditions for imported cases that are difficult to anticipate and that can present at any hospital.

With an active Public Health Emergency of International Concern in place and millions of international visitors converging on North American cities, the longstanding gaps in guidance for laboratory preparedness and testing of persons under investigation (PUI) for Ebola have become increasingly difficult to ignore. Frontline hospitals across the United States are expected to be able to identify, isolate, stabilize, and manage PUIs, who may be critically ill, for up to 72 hours until they can be transferred to a regional designated treatment center (5, 6). Routine diagnostic testing needs to be available during that time, along with Category A shipping capabilities to send samples for viral hemorrhagic fever (VHF) testing. Given current events, many hospitals have had to either create or revisit protocols developed over a decade ago during the 2014 Ebola outbreak, only to find that designated instruments are no longer available, staff are no longer trained, or the original workflow no longer reflects current laboratory operations.

Prior work has documented persistent gaps in laboratory readiness for high-consequence pathogens since the 2014 Ebola outbreak, with many U.S. hospitals remaining unprepared to safely and operationally support laboratory testing for PUIs (5, 7). A 2025 survey of 50 frontline hospitals (48/50 teaching hospitals and 54% tertiary/quaternary care centers) in four major U.S. urban centers found that in the context of a VHF patient, only 64% could safely perform a chemistry panel, and 48% could perform malaria rapid diagnostic testing, despite 88%–90% of respondents rating both as extremely or very important for clinical care (8). Respondents identified multiple obstacles, including inadequate personnel preparation (36%), equipment unavailability (31%), regulatory ambiguity about safe procedures (31%), manufacturer warranty concerns (31%), potential disruption to routine laboratory operations (29%), and unresolved biosafety questions (28%) (8).

We were asked by our institution to evaluate whether our laboratory could manage routine testing for PUIs. Additionally, there was interest in determining whether our core laboratory instruments could be used for Ebola PUI testing as a way to minimize additional instrumentation, training, reagents, and staffing costs. We found that these questions had no straightforward answers.

This report describes our investigation into the instrumentation capabilities, specimen workflow constraints, waste disposal protocols, regulatory requirements, and biosafety infrastructure necessary to safely and effectively process diagnostic specimens from PUIs for Ebola and other select agents. Through a multidisciplinary review of institutional capabilities, external guidance, and operational feasibility, we navigated conflicting literature recommendations and ambiguities in existing regulatory guidance to identify critical gaps in our laboratory’s ability to implement automated instrumentation as recommended by the CDC. Although these findings reflect the experience of a single institution and may vary by hospital infrastructure and state, we believe the operational and regulatory gaps described here are relevant to clinical laboratories across the country.

## LABORATORY INSTRUMENTATION RISK ASSESSMENT

Frontline hospitals across the United States face a constellation of unresolved, sometimes contradictory guidance when attempting to build practical VHF laboratory preparedness workflows. The U.S. Centers for Disease Control and Prevention (CDC) guidance explicitly acknowledges that hospitals have safely used core laboratory instruments to test specimens from PUIs for VHF, an approach that may be more feasible for institutions without dedicated laboratory biocontainment infrastructure (9). Nebraska Medicine’s Biocontainment Unit demonstrated, in the 2014 outbreak, an approach that safely incorporated core laboratory equipment (10). New York (NY) State guidance gives hospitals flexibility to perform point-of-care testing (POCT) outside the patient room, in the laboratory, or use automated instruments consistent with CDC recommendations (6). However, NY State guidance provides substantially more operational detail for POCT infrastructure than for automated laboratory instruments, for which it primarily refers users to CDC guidance (6). The scope and emphasis of these different approaches are illustrated in Table 1. Indeed, hospitals may use different approaches based on their resources and infrastructure (11, 12).

**TABLE 1:** Comparison of laboratory testing approaches for patients with suspected or confirmed VHF across published guidance documents and reported institutional experiences^*a*^

	Guidance documents			Institutional experience			
	CDC (2026) (9)	NY State (2025) (6)	Region 1 RESPTC Toolkit (2024) (13)	Texas Presbyterian Dallas (2014) (14)	Nebraska Medicine (2014) (15)	Emory (2014) (16)	Survey: U.S. Designated ETCs (2016) (12)
Testing location	Core lab or POCT	POCT outside the patient room, in the lab, or in the core lab	Clinical lab only; handheld analyzers only in BSC	Core laboratory + bedside	BSL-3 +space, on-campus public health lab, core lab, POCT	POCT laboratory in an isolation space near the patient room	87% had bedside testing, 94% clinical laboratory, and core lab use not specified
Test menu	Baseline: CBC with diff, chemistry, blood cultures, PT/INR, urinalysis, LFTs, malaria smear, COVID ± influenza/RSV	Comprehensive: CBC with diff, chemistry, coagulation, urinalysis, LFTs, malaria smear, troponin, ± blood cultures, COVID, influenza	Limited: BMP and malaria RDT described^*b*^; smear not recommended^*b*^	Comprehensive, varied by patient location (ED vs. ICU)	Comprehensive: CBC, Chem, blood cultures, coagulation, malaria smear, troponin, blood gases, blood typing + others (no LFTs mentioned)	Limited: CBC, chemistry, INR, urinalysis, blood gases, malaria POC	Not detailed; distributed among multiple locations
BSC requirement	Class II BSC where possible; splash shield + enhanced PPE as alternative	Class II BSC is required in the laboratory	Class II BSC required	Used class II BSC, +high-level PPE for core lab	BSC class not defined, but some testing performed in BSL3 lab	BSC class not defined but present	Dependent on location
Key constraints/notes	Does not provide specimen handling for specific instruments; the relative risks of mitigation strategies are ambiguous	Provides detail for POCT; primarily refers to CDC for automated instruments	Assumes dedicated BSC + infrastructure, direct guidance provided only for a limited test menu	Core lab testing in high-level PPE at fixed times with restricted staff movement; operationally restrictive, staffing anxiety/stress	Required substantial institutional resources and coordination	Required containment infrastructure. No centrifugation used, CBC/Biofire instrument placed next to the hood, 10 trained staff	Survey did not specify which tests were done; snapshot only; institutions made varying choices about the distribution of labor

^*a*^ Ebola/VHF laboratory guidance and institutional experience comparison. BMP, basic metabolic panel; BSC, biosafety cabinet; BSL, biosafety level; CBC, complete blood count; diff, differential; ED, emergency department; ETC, Ebola treatment center; ICU, intensive care unit; INR, international normalized ratio; LFT, liver function test; PHL, public health laboratory; POC/POCT, point-of-care testing; PPE, personal protective equipment; PT, prothrombin time; RDT, rapid diagnostic test; RESPTC, Regional Emerging Special Pathogen Treatment Center.

^*b*^ Other testing may be incorporated, dependent on hospital capability and risk assessment.

In contrast to the above, the Laboratory Testing Toolkit developed at Massachusetts General Hospital, a Region 1 Regional Emerging Special Pathogens Treatment Center (RESPTC), recommends performing all testing within the clinical laboratory using POCT instrumentation operated within a class II Biosafety Cabinet (BSC) under biosafety level 2 laboratory containment with enhanced personal protective equipment (PPE) and safety precautions (13). It also discourages testing at or near the point of care, citing concerns about procedural adherence, competency maintenance, and regulatory oversight, consistent with concerns raised in the published literature (12). The toolkit presumes a dedicated BSC and requires training infrastructure. It also offers a limited test menu, restricted to a basic metabolic panel (BMP) and rapid malaria testing, and states that blood smears should not be performed due to procedural risk. The authors suggest that safely performing additional tests within this framework may require more extensive risk assessment, should laboratories have the capability to do so (13). This contrasts with the minimum testing standard set by NY State guidance, which additionally includes complete blood count (CBC), coagulation studies, and liver function tests (LFTs), and recommends performing a thin smear for malaria as part of the diagnostic workup (6). CDC also recommends performing a blood smear for malaria (9). The gap between what the CDC defines as a minimum standard and what a published toolkit explicitly designed for generalizability across frontline facilities recommends is safely deliverable is difficult for clinical laboratories to navigate as they develop plans for institutions that range from small community hospitals to large urban academic centers.

The use of automated core laboratory instrumentation is operationally appealing because it leverages infrastructure that is already maintained, quality-controlled, familiar to staff, and may require less manual sample manipulation. At our institution, the VHF plan developed more than a decade ago relied primarily on manual blood smears and the point of care Abbott iSTAT (Abbott Point of Care Inc., Princeton, NJ) instrument for basic chemistries (electrolytes, total CO2, blood urea nitrogen, creatinine, glucose, calculated hemoglobin, and hematocrit) and blood gases (pH, pCO2, pO2, O2 saturation, total CO2, and lactate) run by laboratory personnel in a BSL-2 space, with dedicated staff trained on appropriate specimen handling. When this plan was revisited in early 2026, the prior approach was no longer operationally viable. The workflow had been developed as a contingency plan rather than a durable, routinely maintained laboratory workflow, and the instrument, reagents, proficiency testing, and staff competency had not been sustained over time. Hospitals with POCT instruments already in routine clinical use could elect to deploy those instruments for special pathogen testing. However, this approach requires sufficient instrument redundancy and training for staff involved in VHF care. Uncertainty remains about whether instruments used for VHF testing can be safely returned to routine clinical service after decontamination. Our iSTAT instrument was not otherwise used in routine patient care. This highlights a practical limitation of maintaining rarely used, stand-alone preparedness equipment. When feasible and safe, adapting existing laboratory infrastructure is more sustainable than preserving instruments and workflows that are otherwise outside routine use.

While CDC guidance supports core laboratory instrument use, the practical execution of this approach is complicated by a recommended extensive risk assessment framework that places substantial burden on individual institutions. A risk assessment is a systematic evaluation that identifies the hazards of working with potentially infectious agents. It determines the likelihood that laboratory personnel will be exposed to hazardous agents through their work processes and evaluates the consequences of such exposure. The assessment examines the specimen’s complete path through the laboratory and all associated work processes to pinpoint potential exposure risks, including specimen transport routes, sharps handling, potential for splashing or aerosol generation, equipment hazards, BSC operation, decontamination procedures, and waste management. The findings are documented in an exposure control plan that specifies the combination of engineering controls, administrative controls, and PPE needed to mitigate each identified risk while also demonstrating compliance with regulatory guidance on specimen handling and appropriate PPE (9, 14)

Risk assessments must be reassessed frequently, with the introduction of new pathogens, equipment, staff, or methods. Cornish et al. document specific biosafety gaps in core laboratory workflows identified during the 2014 outbreak response, including open specimen tubes on conveyor belts, exposed specimen decapper discharge chutes, centrifugation without sealed buckets or rotors, and noted that staff anxiety and fear had direct operational consequences, including the potential for impaired judgment and lapses in safety procedures (14). They also specifically note that the evidence base around both the risks themselves and specific mitigation measures, including substitution of near-patient testing for core laboratory testing, was limited (14). Prior work has shown that manufacturer recommendations for decontamination vary widely between instruments (17). Navigating these complex risk assessments, with limited data on the relative severity of risk and success of mitigation strategies, and conflicting recommendations from within and among institutions, is a burden that falls disproportionately on hospitals with the fewest resources to act on what they find (7).

During our laboratory risk assessment, we initially focused on using automated core lab instrumentation. That review identified gaps in guidance on PPE, specimen handling, and instrument requirements, which made implementation operationally challenging. We also recognized that the electronic medical record (EMR) used by physicians to order testing might not adequately restrict testing to only approved assays, risking inadvertent orders for unavailable or unsafe procedures.

## PPE, SPECIMEN WORKFLOW, AND BSL2+ HANDLING

We noted that PPE required for direct specimen handling is well described in existing guidance, and secondary containment during transport to the laboratory is clearly recommended (9). We initially explored using our closed core lab automated instrumentation, with our BSL2+ space used for sample preparation and centrifugation steps. However, regulatory guidance did not clearly specify the PPE required for loading and unloading specimens into and out of closed automated core lab instruments. In seeking external guidance, we contacted our regional National Emerging Special Pathogens Training and Education Center (NETEC) representatives, whose available experience was primarily with point-of-care testing workflows rather than automated core laboratory instruments. We also contacted the Occupational Safety and Health Administration (OSHA), which referred us to CDC resources. CDC guidance, in turn, emphasized the need for institutions to perform their own site-specific risk assessment to determine appropriate PPE and specimen-handling practices. This may seem like a narrow operational question, but it carries significant implications. If the lab technologist loading samples onto the instrument requires enhanced PPE, it raises an immediate question about whether other staff working in the same open laboratory space also require protection. This created significant concern and operational uncertainty. Even with reassurance that closed instrumentation could be used safely, the idea of performing visibly heightened containment procedures in a shared workspace caused alarm and anxiety among laboratory staff.

CDC guidance on specimen management also specifies that samples should not travel through high-traffic areas and that pneumatic tube transport should not be used (9). High-throughput core laboratory analyzers are, by design, located in open, high-traffic spaces; their throughput often depends on it. In 2014, the Texas Health Presbyterian Hospital Dallas (Dallas, TX) core laboratory performed testing for three confirmed Ebola patients without a biocontainment unit, using high-level PPE, a closed-system sample automation track with core lab instruments, and testing scheduled at fixed times each day with restricted staff movement during testing periods (14). Clearing an entire core laboratory (which routinely processes hundreds to thousands of specimens per hour) to process a single specimen is not operationally feasible in many institutions. It may also impose risk on other patients by delaying urgent testing unrelated to the PUI, including trauma, massive transfusion, transplant, sepsis, hyperkalemia, stroke, or oncology workflows that depend on rapid access to CBC, chemistry, coagulation, blood bank, and blood gas testing. What protection, if any, is required for staff performing routine work nearby who are not wearing enhanced PPE and have no reason to expect proximity to a high-consequence pathogen? This uncertainty was not unique to our laboratory. During the 2014 outbreak, clinical laboratories were concerned about this same issue, specifically the potential risk of aerosolization from highly automated instruments, and many laboratories declined to test at all as a result (18).

CDC’s laboratory guidance states that the risk of VHF transmission in a clinical laboratory is similar to that of other bloodborne pathogens, and that standard precautions and the OSHA bloodborne pathogen standard should effectively prevent laboratory-acquired infection. The same guidance also recommends practices that go beyond standard bloodborne pathogen precautions: closed tube systems, sealed centrifuge buckets or rotors, and BSC loading and unloading where possible. CDC’s biological risk assessment framework flags aerosolization from procedures such as centrifugation, vortexing, and open handling as a potential laboratory-acquired infection risk requiring mitigation, recommending a class II BSC for specimen manipulation when possible (9).

Ebola is not considered an aerosol-transmissible pathogen under natural conditions, and the risk associated with aerosolization in laboratory settings is hypothetical (9, 19, 20). CDC guidance further notes that when a class II BSC is unavailable for sample preparation, splash shields combined with enhanced PPE may serve as an alternative (9). While this acknowledges the reality that BSC infrastructure is not universally available, it presents an unintended problem. Flexibility is not the same as reassurance. Laboratory staff handling specimens in BSL2+ or BSL3 conditions are typically already wearing enhanced PPE, which provides protection against both splash and aerosol exposure. Whether splash shields with enhanced PPE provide an acceptable alternative to a class II BSC, or instead represent an acceptance of additional procedural risk, must be explicitly evaluated as part of the local laboratory risk assessment. Without clarity on what level of protection is definitively required for this specific pathogen and context, the availability of a lower-tier option risks creating greater uncertainty. Hospitals may prefer to avoid risk altogether rather than accept riskier alternatives. Additionally, laboratories attempting to err on the side of caution may find themselves overusing PPE beyond what may be strictly necessary. More PPE is not always better, as use of unfamiliar PPE or equipment without sufficient training and practice may lead to inadvertent breaches in safe technique (9, 21).

The risk assessment framework allows institutions to ultimately determine which instruments are safe to use and under what conditions. Instrument designs and associated biosafety risks are not uniform, and the degree to which any given platform can be used safely requires a detailed review of each step of the process. CDC does not provide specimen handling recommendations for specific instruments, likely because such guidance would need to account for wide variation in laboratory configurations and could be interpreted as endorsing particular products. There are common themes worth noting, however. Many sample automation systems perform a centrifugation step using unsealed rotors and buckets, a potential hazard that was noted in our risk assessment and by other institutions during preparedness evaluations (22). Coagulation testing may involve manual specimen handling steps, such as tube decapping, aliquoting, open-tube manipulation, or centrifugation, which increase the potential for droplet, splash, or aerosol exposure depending on the instrument and workflow. Our automated hematology instruments are equipped with a slide maker that creates a peripheral smear in a partially open laboratory environment rather than within a completely closed system or BSC, creating potential for specimen contact with ambient air during processing and sharps injury risk from possible slide breakage. Our automated urinalysis analyzer operates with uncapped specimens on an open conveyor belt. Our automated coagulation instrument requires manual liquid waste disposal, requiring staff to open, transport, and empty an uncapped large liquid waste container with potential risk for splash, spill, drip, or environmental contamination.

In 2014, manufacturer guidance on instrument decontamination was variable, with some companies claiming that testing specimens from Ebola patients would void the warranty (17, 23). Today, many large instrument manufacturers provide VHF decontamination protocols for their platforms, which represents meaningful progress. However, these protocols still vary considerably and none are explicitly referenced in or endorsed by existing biosafety guidance. For example, our automated hematology instrument used for performing complete blood counts requires a 20-minute downtime for decontamination, while the chemistry line only requires external decontamination. It is not clear whether these are meaningful differences.

CDC guidance does not indicate whether any specific instrument can or cannot be used; it indicates only that the risk assessment will determine that (9). The risk assessment framework asks institutions to evaluate the probability and consequence of exposure, but for Ebola in a clinical laboratory context, neither parameter is well characterized. The probability of exposure from a specific instrument failure point, unsealed rotor, open decapper chute, slide maker, or manual liquid waste disposal is unknown, as there are no clearly documented cases of laboratory-acquired Ebola from routine clinical diagnostic testing. Laboratorians are unable to uniformly weigh these probabilities against the consequence because the consequence of any Ebola exposure is severe, but the actual transmission risk through these routes in this context has never been quantified. Without this evidence base, risk assessments become institutional judgment calls that vary widely depending on the assessor’s risk tolerance, producing inconsistent outcomes rather than the standardized safety baseline the framework intends.

Many laboratories, like ours, will discover that only some of their closed instruments meet their internal risk assessment standards, ultimately requiring investment in instrumentation outside of their normal workflow. When conducted prospectively and with adequate resources, the risk assessment framework should guide institutions toward safe workflows. In practice, however, many institutions initiate this assessment only in response to an outbreak, emerging threat, or active patient case, at which point discovering that core laboratory instruments cannot actually be used safely leaves little time to implement alternatives, acquire equipment, or train staff.

## WASTE DECONTAMINATION AND DISPOSAL PROTOCOLS

Though federal guidance exists, state-level regulations may further complicate hospitals’ ability to adequately perform testing in PUIs or patients testing positive pending transfer to an appropriate facility. CDC does not provide guidance on the disposal of clinical laboratory effluent (i.e., waste from clinical diagnostic assays) for PUIs. During our risk assessment, however, we discovered that in Massachusetts, existing regulatory frameworks governing biological waste disposal, including the Boston Public Health Commission biological laboratory permitting requirements and specific waste and sewage requirements (24, 25), require sequestration of all laboratory waste streams from testing of patients with suspected or proven Ebola. This requirement cannot be met by many automated instruments, whose effluent waste, which includes both reagent and patient sample, drains directly into central hospital plumbing as part of their standard design. This stands in contrast to the management of other waste generated by a PUI (or positive case), where sequestration of waste streams is standard practice. At our institution, we engaged directly with state public health authorities and regulatory bodies to determine whether these existing frameworks preclude the use of automated laboratory instruments for Ebola PUI testing and were advised to sequester all waste, effectively precluding the use of multiple direct-plumbed core lab automated instruments, despite CDC recommendations. Discussions with our local wastewater authority are ongoing. Similar regulatory ambiguity around laboratory waste disposal likely exists in other states and warrants urgent examination, particularly given current outbreak pressures on frontline hospitals nationwide.

## STAFFING AND TRAINING REQUIREMENTS

The staffing requirements for a laboratory performing testing under BSL-2+ precautions with recommended donning and doffing procedures are extensive. A minimum of two laboratory staff per shift are recommended, one performing hands-on testing and one serving as a dedicated safety monitor, with personnel trained and proficient in donning and doffing before they encounter a PUI (6, 13). Donning and doffing training requires dedicated time, regular practice, and direct observation. Competency cannot be assumed from a single training session. While just-in-time training (preparation of staff immediately before encountering a PUI) is technically possible, staff anxiety and stress during an active case compromise careful technique. Competency requires repeated practice in controlled conditions

To ensure adequate staffing coverage, institutions must either maintain a sufficient number of staff with active training across all shifts or establish an on-call system with dedicated personnel, both of which carry significant scheduling and financial implications. At our institution, we determined that a minimum of 8–10 trained staff members would be required, including supervisory or managerial personnel willing to be called in to perform testing if needed. Additionally, at least one staff member must hold International Air Transport Association (IATA) certification for Category A specimen packaging, required for shipping samples to the CDC if needed. We noted that our capacity to perform Category A specimen transport is limited to Monday through Friday, creating a gap in weekend submissions.

## OUR INSTITUTIONAL APPROACH

Taken together, our risk assessment identified multiple unresolved questions, including the absence of clear PPE guidance for instrument loading, staff anxiety about core laboratory testing, instrument-specific open processing steps that could not be adequately mitigated, and the Massachusetts regulatory requirement to sequester all waste streams, including instrument effluent discharged into central plumbing. This led us to conclude that safe use of our core laboratory automated instruments for Ebola PUI testing could not be implemented without significant unresolved risk at this time.

Because our institution has existing class II BSC infrastructure, we were able to leverage a laboratory-based POCT instrument-based workflow, acknowledging that maintaining a dedicated rarely used workflow is challenging and that sustainability requires institutional commitment. We established a dedicated group of laboratory technologists actively undergoing training in appropriate PPE donning and doffing procedures and in the operation of instruments reserved for PUI testing. Staff participation is voluntary. We prioritized staff autonomy around a high-consequence task, recognizing that mandated participation without staff willingness could compromise adherence to donning/doffing protocols and increase error risk under stress. We also acknowledge that routine staff turnover may make it difficult to maintain adequate coverage indefinitely. At our institution, we are still defining the staffing model needed to sustain this workflow over time. Key implementation considerations include training, competency maintenance, routine staff turnover, and after-hours coverage. Because this workflow is expected to be rarely activated, we will need to determine whether dedicated on-call coverage is necessary or whether a broader cross-trained group of laboratory staff can provide reliable 24/7 coverage.

We have repurposed our class II BSC, normally dedicated to mycology, to accommodate VHF specimens should the need arise. Within it, we have placed a portable Radiometer ABL90 Flex Plus blood gas analyzer (Radiometer America Inc., Brea, California, USA) and a dedicated Abbott iSTAT POCT instrument (Abbott Point of Care Inc., Princeton, NJ) for chemistries. We elected to perform blood gases on our existing ABL90 rather than the iSTAT because this instrument is already interfaced for orders and results to Epic and can be used with the safePICO cap-piercing blood gas syringes to minimize exposure to blood. Blood smears for malaria and manual WBC and platelet estimation will also be performed within the BSC, with the understanding that these provide semi-quantitative rather than fully quantitative assessment. We do not yet have a dedicated hematology POCT instrument, but we plan to acquire one.

Given the preanalytical instability inherent to coagulation testing, we opted for a dedicated Roche CoaguChek (Roche Diagnostics, Indianapolis, IN) instrument for use at the patient’s bedside, recognizing that this workflow requires separate operator training and competency documentation. This instrument is FDA-cleared for quantitative prothrombin time (PT) and international normalized ratio (INR) testing for monitoring warfarin therapy and is included in the New York State guidance document for VHF patient testing (6). Although CoaguChek systems have been used and studied for PT/INR measurement outside routine warfarin monitoring, diagnostic use in this setting would be outside the cleared indication, and performance in non-warfarin coagulopathy states is variable (26, 27). We have also relocated an existing BioFire Torch instrument into our BSL-2+ space and are working toward implementing the BioFire Defense Global Fever Special Pathogens Panel (BioFire Defense, LLC, Salt Lake City, UT). This panel offers rapid detection of multiple VHFs, malaria, and other common causes of fever in returning travelers. Use of this panel allows for rapid VHF testing (within hours of presentation) and reduces our dependence on blood smears, which require greater technical skill and carry a higher risk from slide breakage. We deemed this investment feasible because our institution had access to a redundant BioFire Torch instrument. Although our regional Department of Public Health (DPH) can also perform this testing, we preferred an in-house capability to avoid potential delays in specimen transport or courier transport. A positive result could facilitate rapid transfer to a regional RESPTC hospital. A negative RT-PCR result from a blood specimen collected less than 72 hours after symptom onset does not necessarily exclude VHF, and CDC recommends repeat testing if the patient remains symptomatic after 72 hours (28). CDC also recommends confirmatory testing be sent to CDC regardless of initial results, and ambiguity remains about whether a negative panel result can support rapid rule-out in lower-risk PUIs with clear alternative diagnoses, a question with significant operational implications.

**Fig 1:**
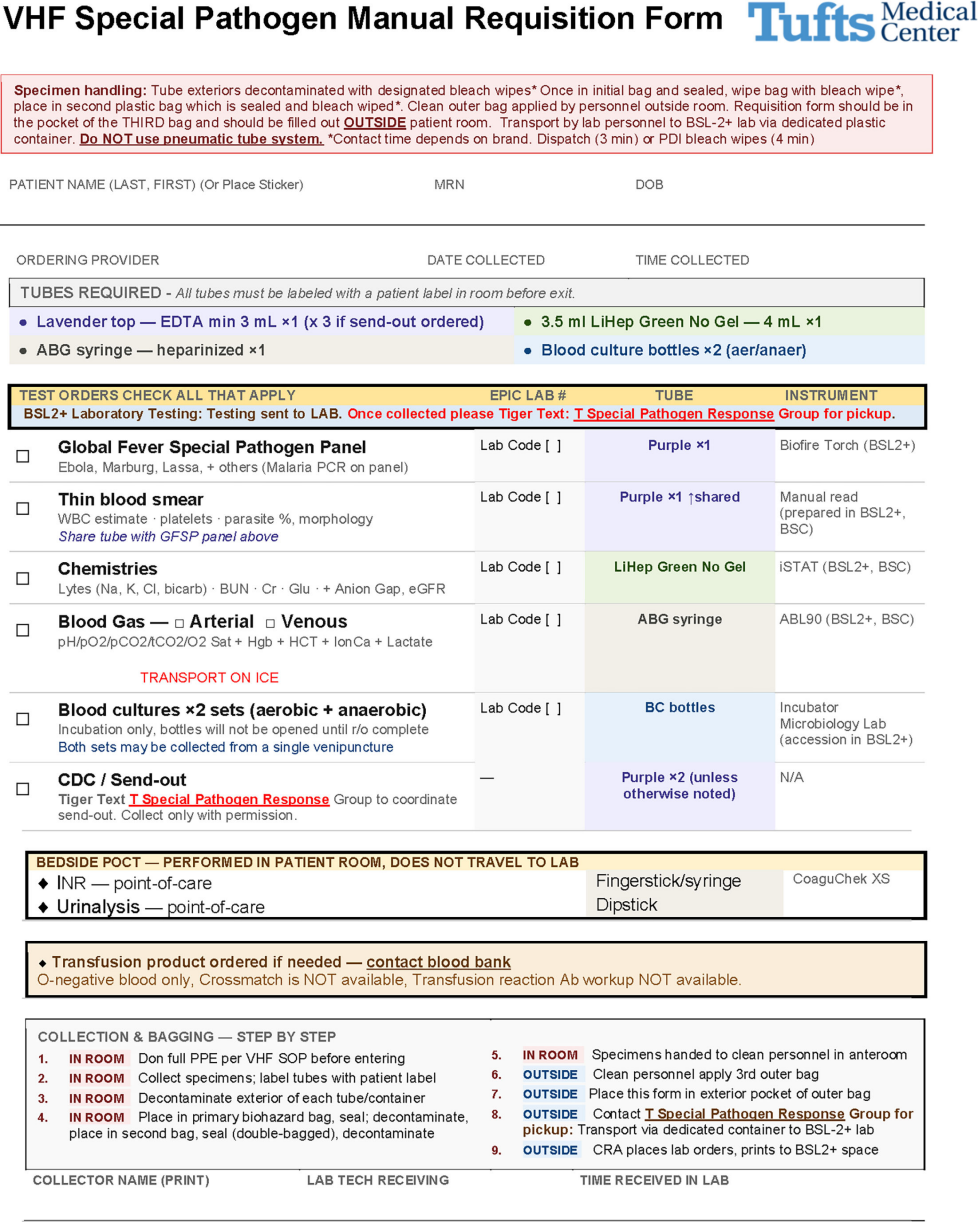
One-page form integrating specimen handling protocols, personnel responsibilities, testing menu with instrument/location assignments, and step-by-step collection workflow. The Global Fever Special Pathogens Panel, if not available on site, would otherwise be performed at the local DPH. The requisition form will be revised to reflect additional hematology POCT capacity when available. Published following departmental review and approval.

To address pre-analytic concerns of test ordering and specimen collection, decontamination, and transport, we developed a paper test requisition form that prevents ordering of unapproved tests and provides step-by-step guidance for specimen handling. Lab staff will transcribe approved test orders from the requisition into the hospital’s EMR using custom test panels built to accommodate our multi-instrument testing approach and to facilitate electronically interfaced results (Fig. 1, draft requisition form).

As an academic tertiary care center, we have the staffing and infrastructure necessary to support the workflows described above. We also serve multiple community hospitals in our network, many of which do not yet have complete laboratory response plans for routine diagnostic testing of possible VHF patients, 24/7 staff with appropriate PPE training, or category A shipping certification.

The question of whether a clinically stable patient should be transferred to another hospital solely for VHF rule-out remains unresolved. Even if transfer is ultimately appropriate, community hospitals may still need to evaluate, isolate, and stabilize patients before transfer can occur. That gap is not theoretical. Recently, a patient returning from Kampala, Uganda, where multiple Ebola cases had been identified, presented to a local community hospital with abdominal pain and vomiting. Due to an initial miscommunication about the patient’s travel itinerary, routine laboratory testing, including CBC, BMP, LFTs, lipase, and urinalysis and imaging studies had already been performed. Only then was the patient isolated and the DPH contacted. The patient was ultimately not deemed a PUI; however, the episode required several hours of multidisciplinary review to determine whether routine clinical care and laboratory testing performed before clarification of the travel history warranted additional exposure assessment, staff notification, or follow-up. In clinical practice, laboratory testing may inadvertently occur before a patient is appropriately recognized as a potential PUI, and the risk to laboratory staff in that context is unclear. These events are likely to occur in the places least prepared to manage them. That is precisely why laboratory preparedness cannot be limited to tertiary centers or specialized referral hospitals.

## COMPLIANCE FRAMEWORK

The expectations placed on frontline hospitals extend well beyond the laboratory. In Massachusetts, the DPH determined that any hospital, including community hospitals, must be capable of caring for a PUI until they are either ruled out or ruled in and can be safely transferred. This requires not only laboratory testing capabilities but also staff, including nursing and physicians, trained and competent in donning and doffing appropriate PPE, and a suitable isolation room with the physical infrastructure to support safe patient care. Appropriate isolation capacity requires physical space, negative pressure capability in some frameworks, and dedicated workflows that do not disrupt the care of other patients. High staff turnover, common across many US hospitals, means that onboarding and training programs must be designed to accommodate a steady influx of new staff who require role-specific training and regular competency assessments (5).

The financial dimension of this problem is not hypothetical either. A 2017 survey of US high-level isolation units found that 14 facilities not designated as RESPTCs collectively spent $9.1 million more than they were reimbursed to develop isolation capabilities and actively struggled to sustain those investments (29, 30). These figures reflect only the costs at the most prepared tier of hospital systems. Hospitals have limited financial incentive to invest in preparedness for low-probability events, despite the potential operational and safety consequences of being unprepared (5).

Confusion over actual risk and appropriate safety measures also impacts patients. During the 2014 Ebola outbreak, a College of American Pathologists (CAP) survey of 28 health systems found that only 4 of 17 responding institutions would allow suspect Ebola specimens into their laboratories at all, and concerns about biosafety contributed directly to delayed diagnoses and patient deaths (14). In April 2016, the Clinical Laboratory Improvement Advisory Committee declared clinical laboratory biosafety an urgent, unmet national need and called upon the federal government to substantially increase biosafety guidance and training for clinical laboratories, a declaration that remains largely unaddressed a decade later (14).

Well-resourced academic and tertiary centers are typically best positioned to sustain laboratory-based POCT-based workflows because they may have dedicated space, robust training and competency programs, and capital for designated instruments. Community hospitals are often less able to maintain the 24/7 staffing, recurrent training, competency, and equipment required for these workflows. Yet the most operationally feasible alternative, use of existing automated laboratory instruments when possible, remains difficult to implement safely without clearer regulatory and biosafety guidance.

Public health authorities have appropriately mandated that frontline hospitals be capable of caring for patients with suspected VHF, but those mandates have not been accompanied by granular guidance on biosafety, laboratory infrastructure, and regulatory clarity necessary to perform laboratory testing in this context. Clearly distinguishing between precautionary measures, evidence-supported requirements, and providing validated examples of sufficient or acceptable mitigation strategies for common instrument configurations would enable meaningful local risk assessments. While these strategies have been described in the published literature, they remain unvalidated and unendorsed by regulatory bodies.

If the expectation is that hospitals unable to use some or all core lab automated instruments solve today’s preparedness gap by purchasing dedicated POCT instruments and establishing class II BSC workflows within BSL-2+-BSL3 environments, the full cost of that solution must be acknowledged: equipment procurement, validation of instrumentation, and ongoing staff competency, including donning and doffing procedures. If the expectation is that standard core laboratory instruments can and should be used, then federal and state authorities must explicitly address instrument design, PPE, wastewater, and regulatory questions. Regardless of which approach institutions pursue, federal and state authorities clarify areas where current guidance diverges or lacks specificity, enabling institutions to confidently choose an appropriate approach for their specific context.

In urban areas with multiple hospitals or among hospital systems with central laboratory infrastructure, consideration should be given to establishing centralized regional facilities capable of performing routine testing services for suspected VHF cases. Inherent limitations, including specimen transport delays and certain time-sensitive testing constraints (e.g., blood gas), would affect feasibility. Still, consolidating expertise and equipment at regional facilities could substantially enable community hospitals to focus on patient identification and stabilization pending transfer while reducing the burden of independently maintaining rarely used infrastructure.

The issues described here are not new. Many surfaced during the 2014 outbreak and have not been resolved in the decade since (14). Still, hospitals are expected to meet preparedness standards that assume levels of infrastructure, staffing, and training most facilities lack and cannot realistically maintain, while also being tasked with independently navigating federal and state regulatory hurdles in the midst of an active outbreak. Ultimately, if frontline hospitals are expected to independently care for and provide basic laboratory testing for VHF PUIs, they need a clear, funded, and practical preparedness framework before a patient arrives.
